# Citrulline Is an Important Biochemical Indicator in Tolerance to Saline and Drought Stresses in Melon

**DOI:** 10.1155/2013/253414

**Published:** 2013-11-30

**Authors:** Sebnem Kusvuran, H. Yildiz Dasgan, Kazim Abak

**Affiliations:** ^1^Kizilirmak Vocational High School, Cankiri Karatekin University, 18100 Cankiri, Turkey; ^2^Horticultural Department, Agricultural Faculty, Cukurova University, 01330 Adana, Turkey

## Abstract

Salt- and drought-induced alterations in citrulline were assessed in 4 local melon genotypes, 2 sensitive (CU-52, CU-94) and 2 tolerant (CU-196, CU-280), grown in vermiculite in a growth chamber. Salt and drought stresses were started using 30-day-old plants, with 250 mM NaCI and 45 mM PEG (−1.0 MPa) and continued for 12 days. After 12 days under salt and drought conditions, the citrulline contents were increased in the tolerant CU 196 to 25.10 **μ**mol gDW^−1^ and 24.10 **μ**mol gDW^−1^ for salt and drought stresses, respectively. However, the citrulline contents of the sensitive CU-52 were 11.68 **μ**mol gDW^−1^ and 11.76 **μ**mol gDW^−1^ for salt and drought, respectively. The striking alteration was obtained in the citrulline accumulation. The tolerant melons accumulated 2 times more citrulline than the sensitive melons. For assessing or screening melon genotypes in a large number of accessions or breeding lines for their tolerance to salinity and drought during their young plant stage, the amount of citrulline accumulation in response to the given treatments might be considered as a novel biochemical indicator of interest in early selection studies.

## 1. Introduction

Abiotic stress is a major cause in limiting the food production worldwide [1–4]. Among the abiotic stresses, salinity and drought dramatically reduce plant growth and productivity, more than any other stress factors [4, 5]. The improvement of stress-tolerant plant cultivars is inevitably important. The local plant genotypes in arid and semiarid regions have become interesting plant material for salinity and drought improvement studies. Plant species and cultivars within a crop species differ greatly in their response to stress. Genetic variability within a species is a valuable tool for screening and breeding for higher tolerance [6, 7]. *Cucumis melo* L. is the first horticultural crop for cultivating in arid and semi-arid regions, where salinity and drought threats are a problem [8].

Drought- and salinity-tolerant plants accumulate organic several osmolytes, especially organic compatible solutes, in response to osmotic stress [9]. The primary function of compatible solutes is to maintain cell turgor and to take up more water from the soil [10]. Compatible solutes fall into 3 major groups: amino acids (e.g., proline), quaternary and tertiary onium compounds (e.g., glycine betaine, dimethylsulfoniopropionate), and polyol/small sugars (e.g., mannitol, trehalose), all of which are substances that are highly soluble in water [9]. The compatible solutes can also act as free-radical scavengers and directly stabilize membranes and/or proteins [10]. Citrulline is a nonessential amino acid that is reported to be an efficient hydroxyl radical scavenger and is a strong antioxidant [11]. Akashi et al. [12] reported that citrulline effectively protects DNA and metabolic enzymes from oxidative injuries. Kawasaki et al. [13] reported that in the Botswana desert, under the conditions of severe water deficit associated with strong sunlight and high temperatures, after the C4 plants have turned yellow, wild watermelon, which is a C3 plant, still remains green and survives for a longer period. Survival under such harsh conditions is temporally associated with the accumulation of the ureido amino acid citrulline in nondamaged leaves. The accumulation of citrulline in response to drought stress in wild watermelon is a unique phenomenon in C3 plants [13]. Akashi et al. [12] and Yokota et al. [14] reported that the accumulated citrulline could contribute to the protection of green tissues from the secondary oxidative stress induced under drought conditions because it behaves *in vitro* as a more potent hydroxyl radical scavenger than compatible solutes like mannitol, proline, and glycine betaine. The citrulline amount actually doubled in CU-196 (tolerant) in comparison to CU-94 (sensitive) under salt stress [15]. Citrulline is a less investigated osmotic solute in drought and salinity than the others.

The main objective of this study was to find out whether citrulline can be used as a biochemical marker of salt and drought stresses in a melon screening studies. In the present work, citrulline accumulation under salinity and drought conditions was compared, separately in tolerant and sensitive melons, with the alterations of proline, total amino acids, and osmotic potentials.

## 2. Materials and Methods

In this study, 4 melon genotypes were used. Salinity and drought responses of the genotypes were previously reported [8, 15]; CU 196 was tolerant, CU 280 was moderately tolerant, and CU-52 and CU-94 were sensitive melons to both salt and drought. Seeds were germinated and the plants were grown in vermiculite in a growth chamber at 26°C/22°C ± 2 day/night temperatures with a light/dark photoperiod of 16 : 8 and photosynthetic photon flux density of 350 *μ*mol m^−2^ s^−1^ at plant height. Relative humidity was maintained at 65 to 70%. The plants were irrigated with half-strength Hoagland's nutrient solution [16]. The composition of the nutrient solution used was as follows (M): Ca(NO_3_)_2_·4H_2_O, 3.0 × 10^−3^; K_2_SO_4_, 0.90 × 10^−3^; MgSO_4_·7H_2_O, 1.0 × 10^−3^; KH_2_PO_4_, 0.2 × 10^−3^; H_3_BO_3_, 1.0 × 10^−5^; 10^−4^ M FeEDTA, MnSO_4_·H_2_O, 1.0 × 10^−6^; CuSO_4_·5H_2_O, 1.0 × 10^−7^; (NH)_6_Mo_7_O_24_·4H_2_O, 1.0 × 10^−8^; ZnSO_4_·7H_2_O, 1 × 10^−6^. The melon plants were 30 days old at the onset of the salt and drought treatments. The experiment was designed as a completely randomized plot with 3 replicates. The salt treatment started with 50 mM NaCl and was then enhanced daily by 50 mM NaCl, up to a final concentration of 250 mM within 5 days. For the drought stress, PEG 6000 (Industrial Chemicals Ltd., Thailand) with 45 mM (−1.0 MPa) concentration was used. The unstressed (control) and stressed plants were harvested at 3-day intervals, at 38, 41, 44, and 47 days after sowing (DAS) for analysis. The melon genotypes were investigated for citrulline, osmotic potential, total amino acid, proline, and shoot dry weight.

For citrulline determination, the solutions were prepared according to Knipp and Vasak [17]. Color developing reagent (COLDER) consists of 1 vol of solution A and 3 vol of solution B. It must always be freshly prepared and should be stored at 4°C in the dark. After its preparation, it should be used within 1 h. Solution A: this solution contained 80 mM DAMO (diacetyl monoxime, 3-hydroxyimino 2-butanone) and 2.0 M TSC (thiosemicarbazide). It was prepared as follows: 1.62 g DAMO and 36 mg TSC were dissolved in 200 mL of H_2_O and stored in the dark at 4°C. The solution should not be used when being older than 1 month [17]. Solution B: this solution contained 3 M H_3_PO_4_, 6 M H_2_SO_4_, and 2 mM NH_4_Fe(SO_4_)_2_ and was prepared as follows: 200 mL of 85% H_3_PO_4_ was slowly added to 450 mL H_2_O while gently stirring. To this solution, 330 mL of 96%–98% H_2_SO_4_ was slowly added. In this mixture, 750 mg of NH_4_Fe(SO_4_)_2_·12H_2_O was dissolved. Upon cooling to room temperature, a final volume of 1 L was carefully adjusted with H_2_O [17].

The citrulline calibration curvewas performed according to the method of Knipp and Vasak [17]. A stock solution of 96 mM citrulline was used. The calibration curve was obtained using citrulline concentrations ranging from 0 to 400 *μ*M in a volume of 60 *μ*L. All of the measurements were performed in duplicate [15]. Subsequently, 200 *μ*L of COLDER was added and a lid was used for closure. The tubes were then incubated for 15 min in a water bath at 95°C and allowed to cool down for 10 min at room temperature. The absorbance of the chromogen was measured within 20 min.

Citrulline extraction from leaves was obtained by homogenizing in 1.5 mL of ethyl alcohol (EtOH) (96%) 500 mg of dry melon leaves. The extracts were heated at 100°C until complete evaporation of the EtOH. The residues were then dissolved in 1.5 mL of cold water and vigorously mixed. After centrifugation of the homogenates (10 min, 5000 g, 24°C), the supernatant (the crude extracts) were removed and stored at −20°C until purification by ion exchange [15].

Fractionation of crude extracts by cation exchanger was performed according to the following. First, a 2 mL column of Dowex 50W-X8(H+) resin (16–40 mesh) was successively washed with 2 mL of H_2_O, 2 mL of 2 N HCI, and 3 mL of H_2_O. Then, the plant extract (300 *μ*L) was loaded onto the column, which was then washed with 5 mL H_2_O to discard sucrose and other neutral substances. The adsorbed citrulline and other amino acids were then eluted with 2 mL 4 M NH_4_OH and 2 mL H_2_O. The eluates were directly used for the measurement of citrulline. Under these conditions, the yield for citrulline fractionation was close to 80% [15].

From the eluates, 600 *μ*L was mixed with 2 mL of COLDER and heated for 15 min at 95°C for measurement of citrulline. After cooling to room temperature, the colored mixtures were transferred into 3 mL quartz cuvettes to measure their absorbance at 540 nm [15].

The osmotic potential was measured using a cryoscopic osmometer (Roebling MROE 01, Bioblock Scientific, France) using leaf extract with fresh tissues according to Trotel et al. [18]. The tissue sap was extracted by centrifuging the homogenized leaf at 5000 rpm for 15 min. The osmotic potential was expressed as MPa.

The total amino acid content was measured from the leaves according to Magne and Larher [19]. The proline content was measured directly on aliquots from the crude extracts of the leaves according to the method of Magne and Larher [19].

For comparison of means, the data were examined statistically using 3-way ANOVA followed by a least significance difference multiple comparison test (*P* < 0.05) which was performed using SAS 9.1 version. Standard deviations were calculated using 3 different plants (replicates).

## 3. Results and Discussion

Salinity and drought increased the citrulline accumulation of the melon genotypes compared to the control plants; however, the responses of the tolerant and sensitive genotypes significantly differed (Table 1).

After 9 days of exposure (44 DAS) to 250 mM NaCI stress, the citrulline levels were almost 2 times higher in the tolerant CU-196 and the moderately tolerant CU-280 genotypes than in the sensitive CU-52 and CU-94 genotypes. After 12 days of exposure (47 DAS) to 250 mM NaCI stress, the citrulline content continued to increase in the tolerant CU-196 genotype, reaching 25.10 *μ*mol gDW^−1^. At the same time in the moderate-tolerant CU-280 genotype, the citrulline concentration was 18.95 *μ*mol gDW^−1^, while the salt-sensitive CU-52 and CU-94 genotypes contained 11.68 and 8.49 *μ*mol gDW^−1^ citrulline, respectively.

Under salt stress, the 4 melon genotypes showed a tendency to increase their citrulline contents. However, the tolerant and moderately tolerant genotypes increased their citrulline contents faster and with higher ratios than the sensitive genotypes. With regard to the citrulline, the melon plants responded faster to drought stress than to salinity. Citrulline accumulation began earlier under drought stress than under salt stress. After 3 days under drought stress, citrulline accumulation had started and gradually increased, and after 9 days, citrulline accumulation was 2 to 4 times higher than in the control plants (Table 1). The important point is that the sensitive CU-52 and CU-94 genotypes seemed to be more responsive to citrulline accumulation under drought stress than under salinity stress.

After 12 days (47 DAS) under drought stress, the citrulline contents in genotypes CU-196 and CU-280 were 24.07 and 19.47 *μ*mol gDW^−1^, respectively (Table 1). On the same day, the drought-sensitive genotypes CU-94 and CU-52 produced 18.52 *μ*mol gDW^−1^ and 11.76 *μ*mol gDW^−1^ citrulline, respectively. The citrulline contents of the melon genotypes increased under salt and drought stresses. However, the tolerant and moderately tolerant CU-196 and CU-280 genotypes accumulated significantly more citrulline than the sensitive CU-94 and CU-52 genotypes.

The greater responsiveness of citrulline metabolism was indicated in the tolerant genotypes. The relationship between citrulline accumulation and tolerance remains to be ascertained because citrulline overproduction might merely be one of the metabolic consequences of adaptation to high saline and drought conditions. Dasgan et al. [15] reported that the salt-tolerant CU-196 melon genotype accumulated more citrulline (57.7 *μ*mol gDW^−1^) than the sensitive CU-94 genotype (22.6 *μ*mol gDW^−1^) after 16 days of exposure to 250 mM NaCI stress. Drought-tolerant wild watermelon accumulates high levels of citrulline under drought stress [13]. Akashi et al. [12] reported that citrulline is a more effective scavenger of hydroxyl radical than mannitol and proline under drought conditions in wild watermelon. Citrulline accumulation in watermelon leaves is an effective defense against oxidative injuries during drought stress [12]. However, it should be noted that citrulline accumulation in the desert plant watermelon growing in Botswana has been shown to be involved in osmotic adjustment, owing to its very high concentration, its compatibility, and its putative protecting properties against oxidative stress through its high potency in scavenging hydroxyl [12]. According to these authors, when wild watermelon was subjected to saline conditions, it overproduced gamma-aminobutyric acid, proline, and glutamine, not citrulline. The reason(s) for such a discrepancy between the metabolic salt responses of *C. lanatus* and that of *C. melo* remain to be elucidated.

Leaf osmotic potential shows the amount of solutes that accumulate in drought- and salinity-stressed plants. The leaf osmotic potential of the melon genotypes generally decreased under stress conditions; however, the responses of the tolerant and sensitive genotypes differed (Table 2). After 12 days (47 DAS) of exposure to 250 mM NaCI stress, the osmotic potential of the tolerant and moderately tolerant CU-196 and CU-280 genotypes was −1.27 MPa and −1.33 MPa, respectively, while the osmotic potential of the sensitive CU-52 and CU-94 genotypes was −1.87 MPa and −2.46 MPa, respectively. After 12 days under drought stress, the leaf osmotic potential of the tolerant and the moderately tolerant CU-196 and CU-280 genotypes was −1.69 MPa and −1.92 MPa, respectively. However, under the same drought conditions, the osmotic potential of the sensitive CU-52 and CU-94 genotypes was −2.03 MPa and −2.16 MPa, respectively (Table 2). The idea considered was that osmotic adjustment, the ability to accumulate solutes in response to salt and water stresses, may contribute to tolerance among melon genotypes. Osmotic potential was generally decreased, as the negative value of MPa, in melon genotypes under salt and drought conditions in comparison to the control. However, this decrease was significantly lower in the sensitive melon genotypes than the tolerant ones. It can be said that the sensitive melon plants tried to adjust their osmotic conditions because of stress response, while the tolerant melon plants had higher osmotic potential and it is possible that they might not have sensed stress at the same time. Even the status of tolerance attributes to a difference in osmotic potential between the moderately tolerant CU280 and tolerant CU196 genotypes (Table 2).

The total amino acid contents in the melon genotypes gradually increased under stress conditions. After 6 days (41 DAS) under salt stress, the total amino acid contents began to increase in the tolerant genotypes (Table 3). After 12 days of exposure to 250 mM NaCI stress, the total amino acid contents of the tolerant and the moderately tolerant CU-196 and CU-280 genotypes were 28.43 *μ*mol gDW^−1^ and 28.75 *μ*mol gDW^−1^, respectively. While under the same saline conditions, the total amino acid contents of the sensitive CU-52 and CU-94 genotypes were 23.94 *μ*mol gDW^−1^ and 16.63 *μ*mol gDW^−1^, respectively. After 12 days under drought stress, the total amino acid contents of the CU-196 and CU-280 genotypes were 27.80 *μ*mol gDW^−1^ and 31.09 *μ*mol gDW^−1^, respectively. While under the same drought conditions, the total amino acid contents of the sensitive CU-52 and CU-94 genotypes were 21.73 *μ*mol gDW^−1^ and 24.56 *μ*mol gDW^−1^, respectively (Table 3). The total amino acid content in the leaves of all of the melon genotypes began to increase after 6 days under salt and drought stress and continued to gradually increase. However, the increase rate was somewhat higher in the tolerant melon genotypes than in the sensitive ones. The tolerant CU-196 melon genotype, even under control conditions, always had a higher total amino acid content. Kawasaki et al. [13] reported that the total amino acid content of wild watermelon gradually increased during exposure to drought stress. The authors indicated that citrulline was a minor component before exposure to stress in wild watermelon, but 3 days after withholding water, citrulline became one of the major amino acids in the total free amino acids. Substantial increases in the amount of proline and other amino acids in salinized plants have been widely reported in [20, 21]. The most common suggestion is that free amino acids contribute to osmotic adjustment by acting as osmolytes. Cuin and Shabala [22] suggested that physiologically relevant concentrations of free amino acids might contribute to plant adaptive responses to salinity by regulating K^+^ transport across the plasma membrane, thus enabling maintenance of an optimal K^+^/Na^+^ ratio as opposed to being merely a symptom of plant damage by stress. Most of the plants species subjected to stress conditions show an accumulation of amino acids. The roles played by accumulated amino acids in plants vary including acting as an osmolyte, regulation of ion transport, and modulating stomatal opening [23].

The increase of proline contents under the saline and drought conditions was less in comparison to the previous parameters. Generally, tolerant genotypes have increased proline under both saline and drought stresses. Proline response of the sensitive genotypes seemed to be higher under drought than salinity (Table 4). After 12 days of exposure to 250 mM NaCI stress, the proline contents of the tolerant and the moderately tolerant CU-196 and CU-280 genotypes were 7.19 *μ*mol gDW^−1^ and 3.15 *μ*mol gDW^−1^, respectively (Table 4). However, in the same saline conditions, the proline contents of the sensitive CU-52 and CU-94 genotypes were 1.70 *μ*mol gDW^−1^ and 1.33 *μ*mol gDW^−1^, respectively. After 12 days under drought stress, the proline contents of the CU-196 and CU-280 genotypes were 6.83 *μ*mol gDW^−1^ and 3.79 *μ*mol gDW^−1^, respectively (Table 3). Under the same drought conditions, the proline contents of the sensitive CU-52 and CU-94 genotypes were 3.79 *μ*mol gDW^−1^ and 3.05 *μ*mol gDW^−1^, respectively. In the present study, the proline response of the melon leaves was very weak in comparison with the citrulline response detected in plants exposed to 250 mM NaCl and drought stresses for 12 days. It was up to 10 times lower, which is quite unusual in bigger plants. Similar results by Dasgan et al. [15] reported that the salt tolerance of the CU-196 genotype could be associated with a cellular environment where citrulline is 10 times more abundant than proline.

As expected, the salinity and drought decreased shoot dry weights in the melon plants (Table 5). The shoot dry weights of the tolerant CU-196 genotype under salt and drought stresses were decreased by 17.7% and 24.5%, respectively. The shoot dry weights of the moderately tolerant CU-280 genotype under salt and drought stresses were decreased by 18.50% and 27.05%, respectively. The shoot dry weights of the sensitive CU-52 genotype under salt and drought stresses were decreased by 29.95% and 27.14%, respectively. The shoot dry weights of the other sensitive CU-94 genotype under salt and drought stresses were decreased by 23.7% and 31.7%, respectively. When the melon genotypes were subjected to saline and drought conditions, their shoot dry weights were reduced. However, the tolerant genotypes were less reduced than the sensitive ones. Our previous studies about salinity and drought effects on melon genotypes indicated that shoot growth differed significantly among the tolerant and sensitive melon genotypes [4, 8, 15].

All the possible interactions in genotypes × stress × time duration experimental design have been investigated and shown under the related tables (Tables 1–5). All the possible interactions were found to be significant.

The correlations between “citrulline” and other parameters under salinity and drought stresses have also been investigated. Under both stresses conditions, in the tolerant and moderately tolerant genotypes the citrulline content was significantly related with osmotic potential, total amino acids, proline, and plant dry weight. However, in sensitive genotypes was not seen a stable relation between the citrulline and other parameters (Tables 6 and 7).

## 4. Conclusion

In conclusion, for assessing or screening melon genotypes in a large number of accessions or breeding lines for their tolerance to salinity and drought during their young plant stage, the amount of citrulline accumulation in response to the given treatments might be considered as a novel biochemical indicator of interest in early selection studies. There are considerable differences between the salt and drought tolerant and sensitive melon genotypes in their physiological responses such as citrulline total amino acid and shoot dry weight.

## Figures and Tables

**Table 1:** Citrulline in the leaves of melon genotypes exposed to 250 mM NaCl salt and 45 mM PEG (−1.0 MPa) drought stresses (*μ*mol gDW^−1^).

Treatment	Genotype	38 DAS, 3 days in stress	41 DAS, 6 days in stress	44 DAS, 9 days in stress	47 DAS, 12 days in stress	Mean of treatments
Control	CU 52	7.81 ± 1.0^g–n^	3.95 ± 0.4^no^	4.34 ± 0.2^m–o^	5.56 ± 1.3^l–o^	7.19
	CU 94	7.93 ± 3.4^g–n^	9.22 ± 5.2^f–l^	6.01 ± 0.6^k–o^	6.68 ± 1.2^h–o^	
	CU 196	6.55 ± 0.8^l–o^	7.63 ± 0.5^g–o^	5.63 ± 0.7^l–o^	6.61 ± 1.9^i–o^	
	CU 280	9.35 ± 1.5^e–l^	8.93 ± 0.3^f–l^	8.97 ± 0.4^f–l^	9.80 ± 0.3^d–l^	

Salt	CU 52	6.37 ± 2.1^j–o^	3.40 ± 5.3^o^	10.78 ± 4.3^d–i^	11.68 ± 1.8^d–g^	11.75
	CU 94	7.75 ± 1.2^g–n^	10.07 ± 6.6^d–k^	10.36 ± 1.8^d–j^	8.49 ± 1.7^g–m^	
	CU 196	7.57 ± 1.1^g–o^	8.45 ± 0.1^g–m^	19.16 ± 8.3^b^	25.10 ± 2.6^a^	
	CU 280	10.98 ± 1.3^d–h^	10.39 ± 1.6^d–j^	18.44 ± 0.3^b^	18.95 ± 0.7^b^	

Drought	CU 52	7.91 ± 2.0^g–n^	13.64 ± 1.3^c–e^	11.34 ± 0.7^d–g^	11.76 ± 0.6^d–g^	15.26
	CU 94	10.70 ± 1.9^d–i^	11.73 ± 2.5^d–g^	21.36 ± 8.5^ab^	18.52 ± 1.3^b^	
	CU 196	12.85 ± 0.9^d–f^	11.73 ± 0.4^d–g^	23.91 ± 7.8^a^	24.07 ± 2.2^a^	
	CU 280	13.76 ± 0.5^cd^	13.64 ± 3.6^c–e^	17.56 ± 3.6^bc^	19.47 ± 1.8^b^	

Mean of times		9.13	9.40	13.16	13.89	

Mean of genotypes		CU 252	CU 94	CU 196	CU 280	

		7.27	10.74	13.27	13.35	

DAS: days after sowing. Data shown were means of 3 independent plants (replicates). Data in the table bearing the same letter are not significant at 5% probability. Differing genotype responses to salt and drought stresses: CU 52 sensitive, CU 94 sensitive, CU 196 tolerant, and CU 280 moderately tolerant.

Significance of interactions:

***Genotype, Treatment, Time, Genotype × Treatment, Genotype × Time, Treatment × Time, Genotype × Treatment × Time.

The a–o letters are showed statistical groups.

**Table 2:** Osmotic potential in the leaves of melon genotypes exposed to 250 mM NaCl salt and 45 mM PEG (−1.0 MPa) drought stresses (−MPa).

Treatment	Genotype	38 DAS, 3 days in stress	41 DAS, 6 days in stress	44 DAS, 9 days in stress	47 DAS, 12 days in stress	Mean of treatments
Control	CU 52	0.80 ± 0.2^q–u^	0.75 ± 0.1^e^	0.79 ± 0.02^s–u^	0.99 ± 0.1^l–s^	0.85
	CU 94	0.74 ± 0.1^u^	0.77 ± 0.1^tu^	0.78 ± 0.01^r–u^	0.97 ± 0.1^f^	
	CU 196	0.85 ± 0.1^q–u^	0.85 ± 0.1^p–u^	0.90 ± 0.02^o–u^	0.93 ± 0.5^f^	
	CU 280	0.86 ± 0.1^p–u^	0.83 ± 0.2^q–u^	0.87 ± 0.1^o–u^	0.95 ± 0.3^m–u^	

Salt	CU 52	1.37 ± 0.4^fg^	1.78 ± 0.1^de^	1.86 ± 0.1^c–e^	1.87 ± 0.2^c–e^	1.52
	CU 94	1.19 ± 0.2^g–l^	2.15 ± 0.2^b^	2.01 ± 0.1^bc^	2.46 ± 0.7^a^	
	CU 196	1.15 ± 0.1^g–n^	1.09 ± 0.2^j–p^	1.24 ± 0.2^f–k^	1.27 ± 0.2^f–j^	
	CU 280	1.18 ± 0.2^g–m^	1.08 ± 0.2^j–p^	1.33 ± 0.2^f–h^	1.33 ± 0.1^f–h^	

Drought	CU 52	1.01 ± 0.2^k–r^	1.10 ± 0.1^h–o^	1.87 ± 0.1^c–e^	2.03 ± 0.0^bc^	1.45
	CU 94	0.84 ± 0.0^q–u^	1.41 ± 0.2^f^	1.32 ± 0.0^f–i^	2.16 ± 0.2^b^	
	CU 196	1.02 ± 0.1^k–q^	1.09 ± 0.2^i–p^	1.95 ± 0.1^b–d^	1.69 ± 0.1^e^	
	CU 280	1.02 ± 0.2^k–q^	1.08 ± 0.1^j–p^	1.74 ± 0.0^de^	1.92 ± 0.1^c–e^	

Mean of times		1.00	1.16	1.39	1.54	

Mean of genotypes		CU 52	CU 94	CU 196	CU 280	

		1.35	1.40	1.17	1.18	

DAS: days after sowing. Data shown were means of 3 independent plants (replicates). Data in the table bearing the same letter are not significant at 5% probability. Differing genotype responses to salt and drought stresses: CU 52 sensitive, CU 94 sensitive, CU 196 tolerant, and CU 280 moderately tolerant.

Significance of interactions:

***Genotype, Treatment, Time, Genotype × Treatment, Genotype × Time, Treatment × Time, Genotype × Treatment × Time.

The a–u letters are showed statistical groups.

**Table 3:** The total amino acid in the leaves of melon genotypes exposed to 250 mM NaCl salt and 45 mM PEG (−1.0 MPa) drought stresses (*μ*mol gDW^−1^).

Treatment	Genotype	38 DAS, 3 days in stress	41 DAS, 6 days in stress	44 DAS, 9 days in stress	47 DAS, 12 days in stress	Mean of treatments
Control	CU 52	10.47 ± 0.1^q–s^	10.22 ± 0.8^rs^	13.42 ± 0.4^m–r^	15.90 ± 0.1^j–o^	14.06
	CU 94	8.48 ± 0.5^s^	11.14 ± 9.9^p–s^	12.23 ± 7.2^n–s^	13.41 ± 1.4^m–r^	
	CU 196	11.43 ± 2.2^o–s^	18.28 ± 8.9^h–l^	19.66 ±1.9^g–k^	22.36 ± 0.1^e–i^	
	CU 280	13.45 ± 1.6^m–r^	11.55 ± 0.7^o–s^	14.55 ± 0.4^l–r^	18.54 ± 1.0^h–l^	

Salt	CU 52	13.45 ± 0.3^m–r^	15.08 ± 0.1^k–q^	15.63 ± 0.3^j–p^	23.94 ± 0.7^c–g^	19.30
	CU 94	9.25 ± 1.5^d^	13.46 ± 6.3^d–f^	15.75 ± 3.5^j–p^	16.63 ± 3.1^j–n^	
	CU 196	17.85 ± 4.6^l–m^	25.59 ± 8.5^b–e^	25.79 ± 3.4^b–e^	28.43 ± 2.0^a–d^	
	CU 280	12.11 ± 0.9^n–s^	20.11 ± 0.4^f–j^	22.82 ± 2.1^e–h^	28.75 ± 2.9^ab^	

Drought	CU 52	19.77 ± 0.4^g–k^	10.23 ± 2.0^rs^	18.57 ± 0.4^h–l^	21.73 ± 1.5^e–i^	21.61
	CU 94	11.23 ± 2.4^o–s^	20.17 ± 4.3^f–j^	23.72 ± 10.5^d–g^	24.56 ± 7.0^b–f^	
	CU 196	11.23 ± 0.4^o–s^	28.95 ± 4.8^ab^	28.51 ± 2.3^a–c^	27.80 ± 4.8^a–d^	
	CU 280	14.58 ± 1.3^l–r^	22.15 ± 6.4^e–i^	24.85 ± 2.7^b–f^	31.09 ± 1.6^a^	

Mean of times		13.67	17.24	19.62	22.75	

Mean of genotypes		CU 52	CU 94	CU 196	CU 280	

		15.54	15.35	22.86	19.54	

DAS: days after sowing. Data shown were means of 3 independent plants (replicates). Data in the table bearing the same letter are not significant at 5% probability. Differing genotype responses to salt and drought stresses: CU 52 sensitive, CU 94 sensitive, CU 196 tolerant, and CU 280 moderately tolerant.

Significance of interactions:

***Genotype, Treatment, Time, Genotype × Time; *Genotype × Treatment; **Genotype × Treatment × Time; ^ns^Treatment × Time.

The a–s letters are showed statistical groups.

**Table 4:** The proline in the leaves of melon genotypes exposed to 250 mM NaCl salt and 45 mM PEG (−1.0 MPa) drought stresses (*μ*mol gDW^−1^).

Treatment	Genotype	38 DAS, 3 days in stress	41 DAS, 6 days in stress	44 DAS, 9 days in stress	47 DAS, 12 days in stress	Mean of treatments
Control	CU 52	0.74 ± 0.1^r^	1.10 ± 0.1^n–r^	1.51 ± 0.2^k–q^	1.58 ± 0.1^k–p^	1.61
	CU 94	1.35 ± 0.2^l–r^	1.02 ± 0.1^p–r^	1.67 ± 0.8^j–o^	1.87 ± 0.1^i–l^	
	CU 196	1.35 ± 0.2^l–r^	1.53 ± 0.1^k–p^	2.84 ± 0.2^d–f^	3.03 ± 0.1^de^	
	CU 280	1.04 ± 0.1^c–e^	1.09 ± 0.1^n–r^	1.75 ± 0.2^j–m^	2.24 ± 0.2^f–j^	

Salt	CU 52	0.90 ± 0.1^d–f^	0.97 ± .02^fqr^	2.12 ± 0.6^g–k^	1.70 ± 0.2^d^	2.19
	CU 94	1.17 ± 0.5^m–r^	1.88 ± 0.1^h–l^	1.56 ± 0.3^k–p^	1.33 ± 0.1^l–r^	
	CU 196	1.32 ± 0.3^l–r^	2.79 ± 0.3^a^	4.18 ± 0.3^a^	7.19 ± 2.0^a^	
	CU 280	1.13 ± 0.2^m–r^	1.06 ± 0.1^o–r^	2.58 ± 0.4^d–g^	3.15 ± 0.1^d^	

Drought	CU 52	1.15 ± 0.1^m–r^	1.03 ± 0.1^p–r^	2.50 ± 0.2^e–h^	3.79 ± 0.2^bc^	2.52
	CU 94	0.81 ± 0.3^r^	1.05 ± 0.1^p–r^	2.41 ± 0.4^f–i^	3.05 ± 0.5^de^	
	CU 196	1.48 ± 0.2^l–q^	2.63 ± 0.2^d–g^	4.41 ± 0.4^b^	6.83 ± 1.8^a^	
	CU 280	1.12 ± 0.2^n–r^	1.71 ± 0.2^j–n^	2.66 ± 0.1^d–g^	3.79 ± 0.4^c^	

Mean of times		1.13	1.49	2.51	3.29	

Mean of genotypes		CU 52	CU 94	CU 196	CU 280	

		1.59	1.60	3.30	1.94	

DAS: days after sowing. Data shown were means of 3 independent plants (replicates). Data in the table bearing the same letter are not significant at 5% probability. Differing genotype responses to salt and drought stresses: CU 52 sensitive, CU 94 sensitive, CU 196 tolerant, and CU 280 moderately tolerant.

Significance of interactions:

***Genotype, Treatment, Time, Genotype × Treatment, Genotype × Time, Treatment × Time, Genotype × Treatment × Time.

The a–r letters are showed statistical groups.

**Table 5:** Shoot dry weight of melon genotypes exposed to 250 mM NaCl salt and 45 mM PEG (−1.0 MPa) drought stresses (g plant^−1^).

Treatment	Genotype	38 DAS, 3 days in stress	41 DAS, 6 days in stress	44 DAS, 9 days in stress	47 DAS, 12 days in stress	Mean of treatments
Control	CU 52	1.14 ± 0.2^k–p^	1.41 ± 0.1^e–i^	1.44 ± 0.2^d–h^	1.57 ± 0.1^c–f^	1.49
	CU 94	1.23 ± 0.3^i–o^	1.23 ± 0.1^i–o^	1.59 ± 0.1^b–e^	1.54 ± 0.2^k–p^	
	CU 196	1.44 ± 0.4^a^	1.62 ± 0.5^b–d^	1.77 ± 0.2^a–c^	1.89 ± 0.1^a^	
	CU 280	1.27 ± 0.4^g–m^	1.44 ± 0.8^d–h^	1.62 ± 0.1^b–d^	1.79 ± 0.2^ab^	

Salt	CU 52	0.91 ± 0.2^qr^	1.02 ± 0.7^p–r^	1.10 ± 0.4^l–q^	1.10 ± 0.2^l–q^	1.21
	CU 94	1.03 ± 0.5^o–r^	1.03 ± 0.2^o–r^	1.07 ± 0.2^m–q^	1.11 ± 0.1^ef^	
	CU 196	1.26 ± 0.5^h–n^	1.31 ± 0.2^g–k^	1.46 ± 0.3^d–g^	1.54 ± 0.2^d–f^	
	CU 280	1.29 ± 0.2^g–l^	1.29 ± 0.2^g–l^	1.46 ± 0.2^d–g^	1.46 ± 0.2^d–g^	

Drought	CU 52	0.98 ± 0.4^p–r^	0.98 ± 0.3^p–r^	1.03 ± 0.1^o–r^	1.14 ± 0.2^k–p^	1.11
	CU 94	0.85 ± 0.3^r^	0.98 ± 0.2^p–r^	1.09 ± 0.3^l–q^	1.05 ± 0.3^n–q^	
	CU 196	1.03 ± 0.5^p–r^	1.31 ± 0.2^g–k^	1.37 ± 0.2^f–j^	1.41 ± 0.2^e–i^	
	CU 280	1.09 ± 0.1^l–q^	1.10 ± 0.3^l–q^	1.17 ± 0.2^j–p^	1.31 ± 0.2^g–k^	

Mean of times		1.12	1.22	1.34	1.40	

Mean of genotypes		CU 52	CU 94	CU 196	CU 280	

		1.14	1.15	1.44	1.35	

DAS: days after sowing. Data shown were means of 3 independent plants (replicates). Data in the table bearing the same letter are not significant at 5% probability. Differing genotype responses to salt and drought stresses: CU 52 sensitive, CU 94 sensitive, CU 196 tolerant, and CU 280 moderately tolerant.

Significance of interactions:

***Genotype, Treatment, Time, Genotype × Treatment, Genotype × Time, Treatment × Time, Genotype × Treatment × Time.

The a–r letters are showed statistical groups.

**Table 6:** Correlations between citrulline and other parameters under salinity stress.

	Osmotic potential	Total amino acids	Proline	Dry weight
CU 52	0.448	0.664	0.860**	0.662
CU 94	0.482	0.528	0.970**	0.044
CU 196	0.932**	0.734*	0.951**	0.992**
CU 280	0.957**	0.769*	0.983**	0.997**

*Indicates the significance at 0.05 probability level.

**Indicates the significance at 0.01 probability level.

**Table 7:** Correlations between citrulline and other parameters under drought stress.

	Osmotic potential	Total amino acids	Proline	Dry weight
CU 52	0.254	0.624	0.161	0.192
CU 94	0.525	0.811**	0.891**	0.900**
CU 196	0.961**	0.486	0.871**	0.693*
CU 280	0.990**	0.872**	0.964**	0.945**

*Indicates the significance at 0.05 probability level.

**Indicates the significance at 0.01 probability level.
